# Efficacy of 25G vitrectomy combined with intrascleral intraocular lens implantation

**DOI:** 10.1097/MD.0000000000021173

**Published:** 2020-07-24

**Authors:** Shasha Luo, Jinjin Yu, Nannan Ding, Yanghao Chen, Zhifeng Wu

**Affiliations:** Department of Ophthalmology, Nanjing Medical University Affiliated Wuxi Second Hospital, Jiangsu Province, China.

**Keywords:** 25-G vitrectomy, clinical efficacy, intraocular lens implantation without capsular support, transscleral intraocular lens implantation

## Abstract

We investigated the clinical efficacy and safety of 25-gauge (G) vitrectomy combined with intrascleral intraocular lens (IOL) implantation. A 25G vitrectomy combined with intrascleral IOL implantation was performed on 39 patients diagnosed with lens dislocation, IOL dislocation, or aphakia. Changes in visual acuity, intraocular pressure (IOP), number of corneal endothelial cells, location of IOL, anatomic success of IOL, recurrence rate of IOL dislocation, and complications were analyzed. One week postoperatively, the IOL was in the centered position in all patients (100%), and 1 month postoperatively, it was centered in 36 patients (92.3%). IOL haptics were exposed under the conjunctiva in one patient (2.6%). Reimplantation of IOL for IOL dislocation was required in two patients (5.1%). Three to six months postoperatively, the IOLs were in the optimum position in 36 patients (92.3%). There were significant differences between the average logarithm of minimal angle of resolution (logMAR) visual acuity at 1 week, 1 month, 3 months, and 6 months postoperatively and that before surgery (*P* < .05). The average IOP at 1 week, 1 month, 3 months, and 6 months postoperatively was significantly lower than the preoperative IOP (*P* < .05). A 25G vitrectomy combined with intrascleral IOL implantation is effective and safe for the treatment of eyes without capsular support.

## 1 Introduction

In conventional cataract surgery, the intraocular lens (IOL) is implanted into the capsular bag of the lens. However, if there is no capsular support, for example, in cataract surgery where the capsular membrane is inadvertently ruptured, or in dislocation of the lens or IOL due to trauma, the capsular membrane cannot support the IOL. In clinical practice, methods such as IOL suspension, anterior chamber IOL, or iris-fixed IOL have been used to address these issues. However, these methods may cause some serious complications such as dislocation of the IOL due to suture breakage during IOL suspension^[1]^; iritis, macular edema, and other complications resulting from anterior chamber IOL^[2]^; pigment loss, pupil distortion and spontaneous iris atrophy with iris-fixed IOL.^[3]^ In recent years, a new surgical method, intrascleral intraocular lens implantation has become popular worldwide. This surgical approach was first proposed by Maggi et al^[4]^ in 1997. In 2008, Agarwal et al^[5]^ made a surgical modification using fibrin glue instead of scleral flap suture. Recent years have seen many modifications and improvements in this field.^[6–16]^ The Ophthalmology Department in Nanjing Medical University Affiliated Wuxi Second Hospital began performing 25-gauge (25G) vitrectomy combined with intrascleral IOL implantation for the treatment of no orinsufficient capsular support since 2014. In this study, we observed and analyzed the changes in efficacy indicators before and after surgery to evaluate the clinical efficacy and safety of the surgical procedure.

## 2 Methods

### 2.1 Patient selection

This study adhered to the tenets of the Helsinki agreement and the ARVO statement. Besides, the study was approved by the ethics committee of Nanjing Medical University in affiliation with the Wuxi Second Hospital. Written informed consent was obtained from all patients after a detailed discussion of the study procedures.

A total of 39 patients, aged 21 to 69 years were examined in the Department of Ophthalmology, Nanjing Medical University Affiliated Wuxi Second Hospital from October 2016 to December 2018. The patient sample comprised 33 men and 6 women. Patients with a normal cornea and fundus, and those diagnosed with lens dislocation, IOL dislocation, and postoperative aphakia were included in the study. Patients were excluded from the study if they had any of the following exclusion criteria:

1. Severe systemic disease and poor compliance;
2. Serious corneal scars or serious damage to the fundus, which could potentially affect recovery of postoperative visual acuity;
3. A history of other eye diseases, such as scleritis, scleral staphyloma, retinal or choroidal detachment, eye tumor, or optic nerve atrophy.

### 2.2 Preoperative preparation

Each patient underwent the following procedures before study enrolment: visual acuity examination, intraocular pressure (IOP) measurement, slit lamp microscopy, dilated fundus examination, corneal endothelium examination, hexagonal cell ratio measurement, anterior segment photography, fundus photography, B-scan ultrasound, optical coherence tomography (OCT), intraocular lens power measurement, and refractive status examination. All patients had a clear cornea except for those with high intraocular pressure corneal edema. The number of corneal endothelial cells and proportion of hexagonal cells were within the reference range, and fundus examination showed no obvious retinal detachment. Routine protocols with regard to preoperative antibiotics were followed. A written informed consent for surgery was obtained from all patients. After routine skin preparation and irrigation of the conjunctival sac, 25G vitrectomy combined with intrascleral IOL implantation was performed.

### 2.3 Surgical technique

All the surgeries were performed by the same surgeon with an extensive cataract and eye fundus surgery experience (had independently completed more than 3000 cases of cataract combined with vitrectomy). Full mydriasis was achieved before the surgery, and retrobulbar anesthesia was administered with local anesthetic. The surgery was performed using phacoemulsification vitrectomy cutting machine (CONSTELLATION vision system), With intraoperative adjustment of cutting speed, vacuum pressure of 350 mmHg, and perfusion bottle height of about 30 to 50 cm, the intraoperative intraocular pressure was maintained between 20 and 30 mmHg, and the perfusion pressure dropped to 10 to 15 mmHg by the end of surgery. All intraocular lenses used during surgery were 3-piece foldable AMO IOLs (model: AR40e). For patients with lens dislocation and vitreous hemorrhage due to various causes, lens capsule extraction was done initially followed by 25G vitrectomy combined with intrascleral IOL implantation; for patients with dislocation of the IOL, after removal of the lens, 25G vitrectomy combined with intrascleral IOL implantation was performed; for patients with traumatic aphakia who had undergone 25G channel perfusion, anterior vitrectomy followed by intrascleral IOL implantation was performed. Here, we describe the surgical procedure using an aphakic patient as an example.

Take the right eye for example, 25G intraocular perfusion was placed in the flat part of the ciliary body at 7 o’clock positions below the temporal area, 4 × 5 mm conjunctival flap based on the fornix was made at 3 and 9 o’clock positions along the limbus of cornea respectively. Electrocoagulation was used to stop bleeding, and a 3 × 4 mm lamellar scleral flap was created under the conjunctival flap at 3 and 9 o’clock positions. A three channel 25G anterior vitrectomy was performed by puncturing the vitreous with a 25G puncture needle 2 mm behind the lamellar scleral flap at 3 and 9 o’clock positions. The corneal incision was fashioned with a 2.8 mm keratome, and it was slightly enlarged to allow easy insertion of the IOL. The haptic tip was clamped with a 25G intraocular forceps at the 3 o’clock position and then grasped and pulled with the first forceps, thereby externalizing it from the sclerotomy site. The trailing haptic was grasped with forceps and flexed into the anterior chamber. In the same manner, at the 9 o’clock position, the trailing haptic was pulled out of the sclera using the handshake technique,^[9]^ and the position of the IOL was adjusted. Scleral tunnels were placed in the scleralflaps at the 3 o’clock and 9 o’clock positions with a 27G needle under the sclera and the haptics were inserted into the scleral tunnels. The scleral flap and conjunctival incision was sutured using 8–0 suture.

The vitreous perfusion at the 7 o’clock position was stopped and sutured, then the perfusion system was removed. Postoperatively, the puncture hole was carefully checked for any leakage through the puncture port in the conjunctiva. No obvious leakage was observed, and the intraocular pressure was normal. Tobramycin dexamethasone eye ointment was applied and the eye was bandaged. There was no requirement for a special postoperative position. After the surgery, tobramycin dexamethasone eye drops, pranoprofen, and sodium hyaluronate eye drops 4 times a day were prescribed. A close watch was maintained over eye vision, intraocular pressure, intraocular lens position, and development of inflammation and other complications. The patients were followed-up for 3 to 9 months after surgery (average follow-up time 6.23 ± 3.07 months). Visual acuity and IOP measurements, slit lamp microscopy, optometry, observation of fundus and corneal endothelium, ultrasound biomicroscopy (UBM), etc. were performed at 1 week, 1 month, 3 months, and 6 months after surgery.

### 2.4 Statistical analysis

Statistical analysis of all the variables including visual acuity, IOP, corneal endothelium, and hexagonal cell ratios before surgery, 1 week, 1 month, 3 months, and 6 months after surgery, was performed using SPSS 18.0 statistical software. Both the Quantile-Quantile plot and Kolmogorov-Smirnov test (*P* > .05) showed a normal distribution for parameters such as visual acuity, corneal endothelial cells, and intraocular pressure. Statistical analysis of the preoperative and postoperative data was performed using the paired *t* test. Statistical analysis among multiple groups was performed using analysis of variance. *P* < .05 was considered statistically significant.

Vision was converted to the logarithm of the minimum angle of resolution (logMAR) vision to facilitate statistical analysis. The standard logMAR visual acuity with hand motion and finger count is 2.4 and 2.1, respectively.^[10]^

### 2.5 Data availability

The datasets generated during and/or analyzed during the current study are available from the corresponding author on reasonable request.

## 3 Results

### 3.1 Demographic and clinical data

A total of 39 patients, aged 21 to 69 years were studied. The average age of the patients was (54.08 ± 13.42) years. Out of the 39 patients, 33 were male and 6 were female. The demographic data, clinical diagnosis and associated complications are described in Table 1.

**Table 1:**
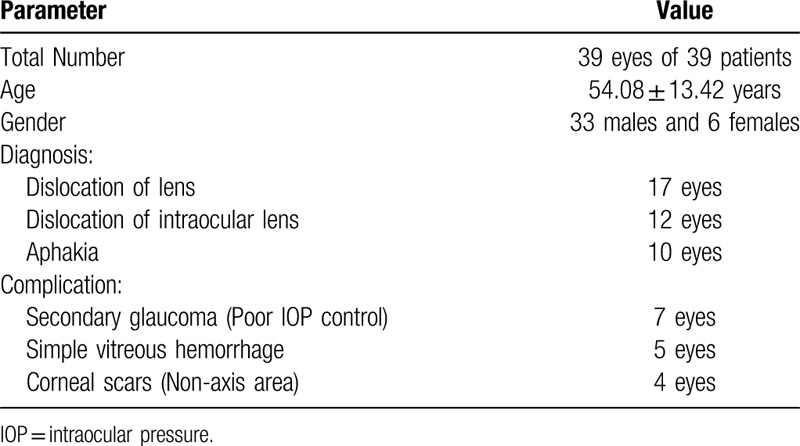
Demographic and clinical data.

### 3.2 Visual outcome

As shown in Table 2, the average best-corrected logMAR visual acuity at 1 week, 1 month, 3 months, and 6 months after surgery was significantly better compared with preoperative values (*P* < .05, *t* values were: 9.70, 11.25, 12.45, 13.26). The patient's visual acuity improved at each time point.

**Table 2:**
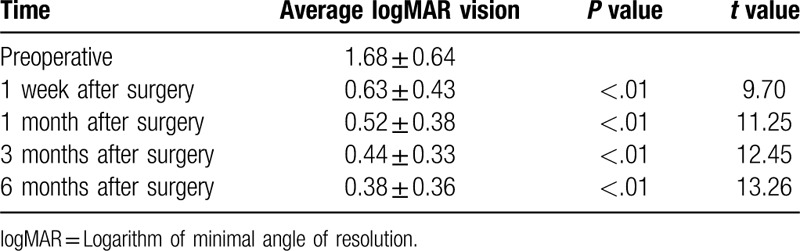
Changes in vision at various times after surgery.

### 3.3 Intraocular pressure

As shown in Table 3, the mean IOP showed significant reduction between the measurements at 1 week, 1 month, 3 months, and 6 months after surgery; and those preoperatively (*P* < .05, *t* values were: 3.14, 3.29, 3.76, 3.32). However, there was no statistically significant difference between IOP measurements at 1 week, 1 month, 3 months, and 6 months (*P* = .902, F = 0.262). Postoperatively the IOP was stable at all-time points. From 1 day to 1 month after surgery, the IOP in 5 patients was < 10 mmHg, and in 3 patients was > 21 mmHg. IOPs were within normal range in all patients after one month.

**Table 3:**
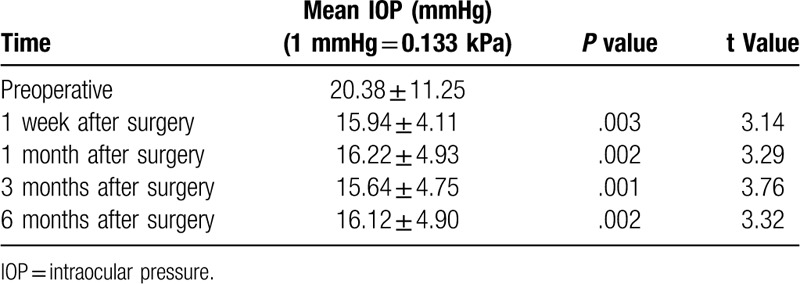
Changes in IOP at various times after surgery.

### 3.4 Number of corneal endothelial cells and mean hexagonal cell ratio

As shown in Table 4, the average number of corneal endothelial cells and the average hexagonal cell ratio 6 months after surgery were significantly different compared to those before surgery (*P* < .05, *t* values: 10.25, 3.90). The surgical approach probably has an effect on the corneal endothelium.

**Table 4:**
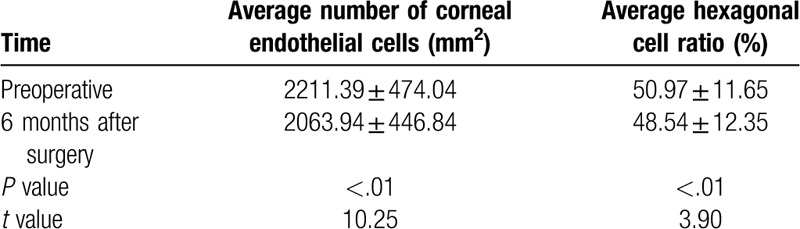
Corneal endothelial changes 6 months after surgery.

### 3.5 IOL positions and complications

**Figure 1:**
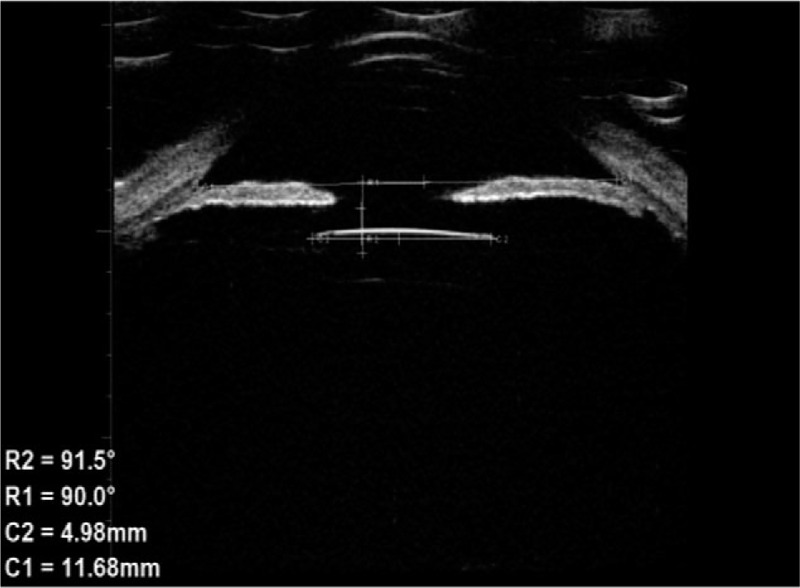
IOL tilt measurement. The angle between the iris–cornea line and the horizontal axis of IOL was calculated as the IOL tilt.

**Figure 2:**
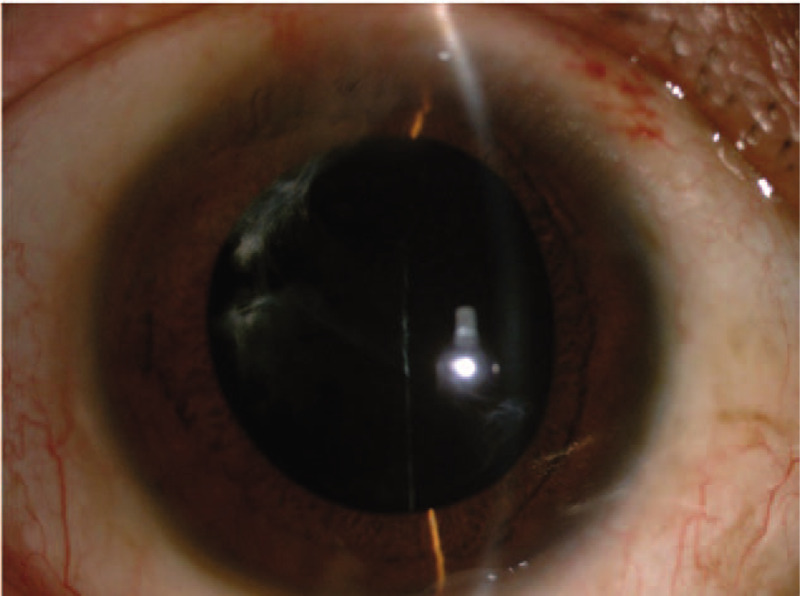
Postoperative IOL position. The position of the intraocular lens was centered 3 months to 6 months after the surgery.

At 1 week after the surgery, all IOLs were in the centered position of the eyes (100%). At the end of the first month after surgery, the IOL was in the centered position in 36 eyes (92.3%), one side of the haptic was exposed under the conjunctiva in one eye (2.6%), and 2 eyes (5.1%) required reimplantation of IOL for IOL dislocation. Between 3 and 6 months after surgery, we used the method of Yamane et al^[7]^ to objectively evaluate the position and measure the tilt of the IOL (Fig. 1). Measurements were made using the UBM, and the angle between the iris–cornea line and the horizontal axis of IOL was calculated as the IOL tilt; the average tilt in this study was 2.27°±2.15. The position of the intraocular lens was centred 3 months to 6 months after the surgery (Figs. 1 and 2) in 36 eyes (92.3%). In the early postoperative period (1 day to 1 month), the major complications (shown in Table 5) were corneal edema and hypotony (23.1% and 12.8%, respectively). Late complications (>1 month) included intraocular lens dislocations, which occurred in 2 cases (5.1%).

**Table 5:**
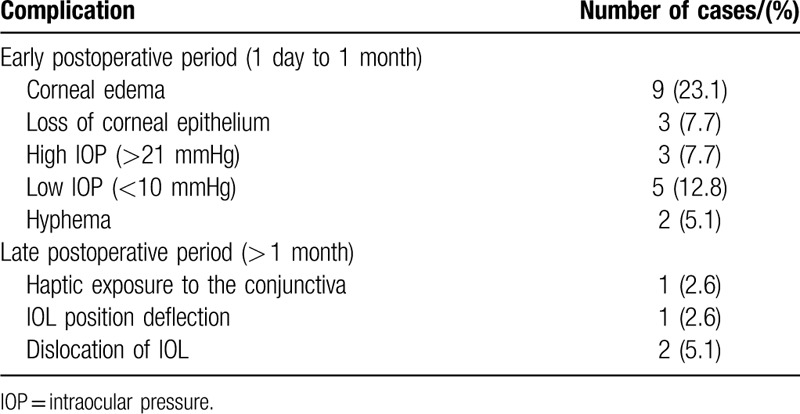
Complications.

## 4 Discussion

Intraocular lens fixation in patients with no capsular support has always been the focus of intraocular lens implantation studies in view of its difficulty. Current surgical methods such as suspended intraocular lens implantation, anterior chamber intraocularlenses (ACIOLs), and iris-fixated posterior chamberintraocular lenses are associated with serious complications.^[1–3]^ In recent years, the internationally popular surgery, intrascleral intraocular lens implantation, has gained usage and has the advantage of being precise and effective with fewer complications. As improved surgical methods have continuously emerged,^[9–16]^ surgery has become simpler, and the surgeon's learning curve has shortened. Our hospital has achieved accurate results since 2014 using 25G vitrectomy combined with intrascleral intraocular lens implantation.

In this study, the difference between preoperative and 1 week, 1 month, 3 months and 6 months postoperative best corrected logMAR visual acuity was significantly different (*P* < .05). The best-corrected logMAR vision at each time point increased by more than 0.2 logMAR units compared to preoperative values, indicating that the visual acuity was improved at each time point.^[10]^ Preoperatively, there were 7 patients with secondary glaucoma caused by dislocation of the lens or IOL. The IOP was high due to obstruction of aqueous humor (average preoperative intraocular pressure was 20.38 ± 11.25 mmHg). After surgery, the IOP was significantly decreased at all follow-ups. The mean IOP at 1 week, 1 month, 3 months, and 6 months after surgery was significantly lower than that before surgery (*P* < .05). This could be because, after surgery, the dislocation of the lens or IOL is corrected and the aqueous humor circulation returns to normal, therefore, the IOP is restored to normal. There was no significant difference between the IOPs at 1 week, 1 month, 3 months, and 6 months postoperatively (*P* > .05), indicating IOP control at all-time points after surgery. The average number of corneal endothelial cells and the mean hexagonal cell ratio 6 months after surgery were significantly different compared to those before surgery (*P* < .05), indicating that the surgical method had a certain influence on the corneal endothelium, which is consistent with previous studies.^[11]^ This also suggests that the surgeon should minimize intracameral surgeries in order to avoid excessive interference with the inner eye, improve proficiency in surgical techniques, and shorten the duration of surgery. In our study, IOLs were in position and centered in 100%, 92.3%, and 92.3% patients after 1 week, 1 month, and 3 to 6 months, respectively. We used the method of Yamane et al^[7]^ to measure the intraocular lens tilt; the tilt was 2.27°±2.15° in our study, which is similar to the results of Yamane et al. The only difference between our study and theirs’ was the fact that their study used anterior OCT to measure the inclination, while in our hospital, due to certain limitations, UBM test results were used to measure the exact inclination.

In terms of complications, minimal bleeding from the anterior chamber may be sometimes being experienced during the surgery. The 25G channel is too close to the limbus, and therefore, the bleeding may be caused by intraocular contact with the root of the iris. This was resolved with appropriate measures. Corneal epithelial edema and loss of corneal epithelium occurred within 1 month after surgery, which was related to the operation. Five patients developed low intraocular pressure (<10 mmHg) within 5 weeks, which may be related to leakage from the incision site. During surgery, we used a 25G vitrectomy channel to pull the 2 haptics of the IOLs out of the sclera, and then combined the scleral flap and the scleral tunnel to fix the 2 haptics. The outer diameter of the 25G channel was 0.5 mm, which is not enough to wrap the 0.33 mm haptic tightly; this may have caused leakage from the incision site, which in turn would have led to low IOP. Although Yamane et al used the 27G sleeve method, they experienced the problem of low IOP too.^[7]^ Subconjunctival haptic exposure and positional deviations of the IOL have also been reported in literature.^[12]^ In our study, after a simple treatment involving fixing the haptics in the scleral layer, the IOL positions were stable and centered. Two patients had recurrent IOL dislocation, which may have been related to the patient resuming physical activity early in the postoperative period. In addition, both patients were early cases and may be related to the surgeon's proficiency and surgical experience. Both patients underwent repeat surgery with IOL fixation.

In terms of surgical experience, we believe that the best distance between the sclera or scleral tunnel and the limbus is 2 mm. A scleral flap too close to the limbus can lead to rupture of the iris root, anterior chamber hemorrhage and other intraoperative complications. If the scleral flap is too far from the limbus, it will result in the IOL becoming relatively short, and the hernia cannot be firmly inserted into the scleral tunnel; this would lead to an unstable intraocular lens position and even dislocation. There are two types of scleral flaps and scleral tunnels covering the IOL haptic. Our research team believes that best method is a combination of the two. This method completely covers the IOL haptic and also firmly fixes the hernia between the scleral layers. The process of pulling the second haptic out of the sclera during the surgery is the most critical and the most difficult, with the need to use intraocular forceps to “handshake” the haptic in the eye.^[9]^ The top of the second haptic is smoothly clamped by the forceps, and is brought out of the sclera in the direction of the haptic. During the process, attention needs to be paid to the possibility of deformation and breakage of the haptic. The thickness of the sclera in which the haptic is implanted should be sufficient. Too shallow will cause the haptic to be exposed under the conjunctiva. Too deep will injure the choroid and cause serious consequences. Half the scleral thickness is generally used to implant the haptic.

In terms of experience, the skill of the surgeon is very important as precise techniques are required for this surgery. The surgeon should be experienced in performing surgeries of the posterior segment of the eye and cataract surgery, and should be able to skillfully use intraocular devices such as intraocular forceps.

With IOL scleral fixation gaining popularity, some new improvements have emerged internationally, such as suture less 27-gauge needle-guided intrascleral intraocular lens implantation with lamellar scleral dissection,^[7]^ and improved self-made 30G catheter traction of artificial lens scleral fixation.^[13–14]^ In 2017, Yamane et al.^[15]^ reported a new intraocular lens implantation technique. Walsh et al^[16]^ modified the technique of Shin Yamane et al, using a 27G trocar. The emergence of various improved surgical techniques has improved maneuverability and made the technique simpler.

In summary, 25G vitrectomy combined with IOL scleral fixation is effective for eyes with inadequate capsular support. Postoperatively, patients have good recovery of vision, stable intraocular lens position, less irritation to the anterior chamber, and relatively lower postoperative complications. However, a relatively small sample size and short follow-up time were some of the limitations of our study. Large-sample, multi-center, long-term follow-up studies are still needed to effectively assess the efficacy and long-term complications.

## Author contributions

Performed the experiment, analyzed the data, drafted the paper, and prepared figures and tables (SSL, JJY); collected and analyzed the data (NND and YHC); designed the experiments and drafted wrote the paper (ZFW).

**Data curation:** Nannan Ding.

**Writing – original draft:** Jinjin Yu.

**Writing – review & editing:** Shasha Luo.
